# The diagnostic value of high-resolution 3D T2-weighted MRI in preoperative evaluation and classification of Herlyn-Werner-Wunderlich syndrome: a prospective study

**DOI:** 10.1186/s13244-026-02405-2

**Published:** 2026-10-09

**Authors:** Yafei Qi, Shuang Song, Xin Gao, Shu Wang, Yiwei Zhang, Yonglan He, Peng Zhang, Menglin Huang, Huadan Xue, Lan Zhu

**Affiliations:** 1 Department of Radiology, Peking Union Medical College Hospital, Chinese Academy of Medical Sciences, Beijing, China; 2 Department of Obstetrics and Gynecology, Peking Union Medical College Hospital, Chinese Academy of Medical Sciences, Beijing, China

**Keywords:** Herlyn-Werner-Wunderlich syndrome, Magnetic resonance imaging, Imaging (three-dimensional), Mullerian ducts

## Abstract

**Objective:**

Herlyn-Werner-Wunderlich syndrome (HWWS) is a rare female reproductive tract malformation. Accurate preoperative diagnosis and classification are vital for surgical planning. This study aims to evaluate the diagnostic value of high-resolution three-dimensional (3D) T2-weighted MRI in the preoperative assessment and classification of HWWS.

**Materials and methods:**

The study prospectively enrolled 84 patients clinically diagnosed with HWWS between May 2020 and January 2025. Seventy patients underwent pelvic MRI examinations, including high-resolution 3D T2 sequences (1 mm slice thickness) and conventional T2 TSE sequences, supplemented by large-field-of-view (FOV) T2 coronal imaging for urinary system evaluation and full-spine X-ray examinations for skeletal assessment. Two experienced radiologists independently evaluated the imaging features in a blinded manner, focusing on uterine morphology, obstruction sites, presence of communications, and related anomalies and complications. Consistency analysis was performed between imaging findings and surgical results in 52 surgical patients.

**Results:**

Uterine morphology included: didelphys (54/70, 77.1%), complete septate (14/70, 20%), and bicornuate uterus (2/70, 2.9%). Among 52 surgical patients, 31 (59.6%) had vaginal-level obstruction with 18 (34.6%) Type I, 11 (21.2%) Type II, and 2 (3.8%) Type III, and 21 (40.4%) had cervical-level obstruction (Type IV and mixed). 3D T2 sequences identified 7 (63.6%) vaginal septum fistulas, outperforming TSE sequences, which detected none (0%) (*p* = 0.005). Associated findings were ipsilateral renal agenesis (98.6%), ovarian endometriosis (22.9%), hematosalpinx (22.9%), and spinal abnormalities (scoliosis (25.7%), mild scoliosis (41.4%).

**Conclusion:**

High-resolution 3D T2 MRI is superior to conventional T2 TSE sequences in the preoperative evaluation of HWWS, especially for identifying communications and precisely locating obstruction sites.

**Key Points:**

***Question*** Accurate preoperative structural assessment and classification of HWWS are clinically essential, yet conventional MRI has limitations in identifying fine anatomical structures and small communications.

***Findings*** High-resolution 3D T2 MRI outperforms conventional T2 TSE in identifying vaginal and cervical communications and precisely localizing obstruction sites in preoperative evaluation of HWWS.

***Critical relevance statement*** This study supports routine inclusion of 3D sequences in HWWS imaging protocols. Combined with renal and spinal assessment, it provides a comprehensive preoperative evaluation that enhances surgical planning precision and optimizes patient management strategies.

**Graphical Abstract:**

**Figure:**
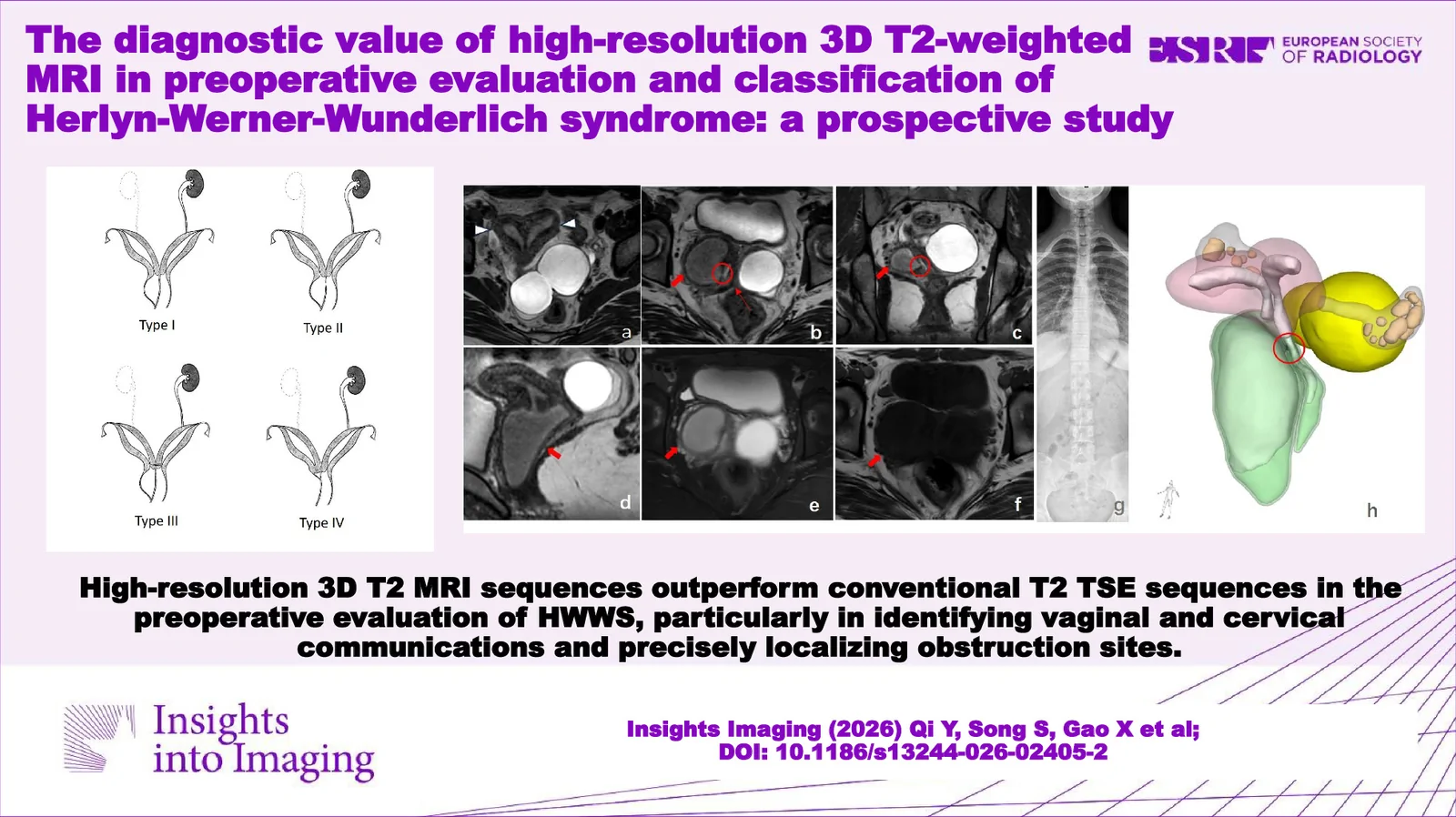

## Introduction

Herlyn-Werner-Wunderlich (HWW) syndrome is a rare and special malformation of the female reproductive system, with an incidence rate of 0.1% to 3.8% [1]; it was first reported by Purslow in 1922, and further reported by Herlyn and Werner (in 1971) as well as Wunderlich (in 1976) regarding related malformations [2, 3], and is named as Herlyn-Werner-Wunderlich Syndrome (HWWS) [4–7]. HWWS is an asymmetric malformation caused by the abnormal development of the Müllerian ducts and the mesonephric ducts during the embryonic period, with the typical manifestations being a double uterus, an oblique vaginal septum, and renal agenesis on the side of the septum [8, 9]. It is also named as Obstructed Hemivagina and Ipsilateral Renal Anomaly (OHVIRA) syndrome and oblique vaginal septum syndrome (OVSS). Most patients with HWWS seek medical attention after the onset of menstruation, often presenting with dysmenorrhea, irregular bleeding, abnormal vaginal discharge, pelvic masses, etc [10]. Early and accurate diagnosis and timely surgical treatment can effectively alleviate obstructive symptoms and reduce the occurrence of complications such as endometriosis and pelvic adhesions [4, 6, 7].

Ultrasonography serves as the first-line imaging modality for HWWS due to its accessibility, real-time capability, and cost-effectiveness [11, 12]. However, its diagnostic capability is limited by operator dependence, a limited field of view, and anatomic distortion from hematocolpos and poor detection of small communications/accurate obstruction level localization [13, 14]. MRI achieves superior accuracy (AUC 0.92 vs 0.88) with multiplanar capability and simultaneous renal evaluation [15], establishing its role as the preoperative gold standard. It offers excellent soft tissue resolution and a broad field of view, allowing for the assessment of uterine anomalies, hematometra, hematocolpos, endometriosis in the ovaries or pelvic region, and kidneys [16]. Communication may exist between the obstructed and the contralateral side. However, conventional T2-weighted TSE MRI exhibits limited sensitivity in detecting cervicovaginal communications, particularly small fistulous tracts traversing the vaginal septum [17]. The T2-weighted turbo spin echo (T2W TSE) sequence is currently mainly used for assessing the structure of the reproductive tract with a thickness of 3–5 mm, while the T1-weighted turbo spin echo (T1W TSE) sequence is utilized to determine the presence of hematocolpos or hematometra [18, 19].

High-resolution 3D thin-slice MRI sequences can provide a resolution of 1 mm for the evaluation of pelvic structures [20]. High-resolution 3D thin-slice MRI sequences have been proven to be applicable for the evaluation of uterine scar and cesarean scar diverticulum [21]. 3D reconstruction technology is also widely used in obstetrics and gynecology. Fan’s study showed 3D MRI of fibroids gave similar volume and bladder-distance measurements as 2D but offered superior spatial detail, aiding treatment planning [22]. Reck-Burneo used 3D cloacography to predict common-channel and urethral lengths for cloacal repair [23]. Three-dimensional MRI may provide more accurate and detailed anatomical information and spatial relationship data in Mullerian duct anomalies [24]. At present, there is a limited amount of research on MRI assessment of HWWS, which is primarily composed of reviews and small sample size descriptive studies. This study prospectively investigates the value of 3D high-resolution thin-slice MRI in the diagnosis and classification of HWWS preoperatively, based on a large sample size, and innovatively applies 3D reconstruction technology for HWWS patients to assess its diagnostic value.

## Materials and methods

**Fig. 1:**
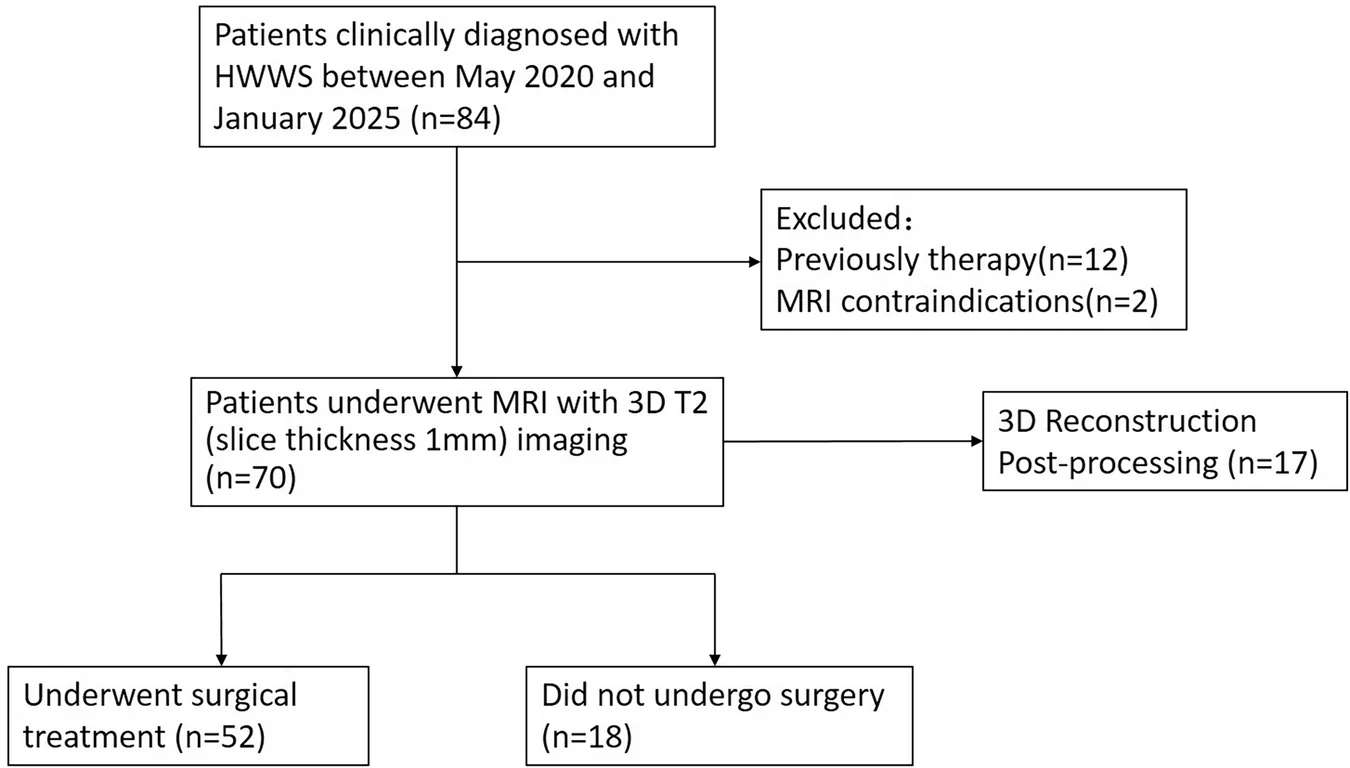
Flowchart of patient selection

This prospective study was approved by the Institutional Review Board of Peking Union Medical College Hospital (IRB Approval I-24PJ1823) and complied with the ethics committee standards. Written informed consent was obtained from all individual participants included in the study. Patients clinically diagnosed with HWWS between May 2020 and January 2025 were included in this study. Patients who had undergone therapy and patients with MRI contraindications were excluded. Finally, 70 patients underwent MRI and were included in the study (Fig. 1).

### Clinical data collection

Clinical data were collected prospectively at the time of enrollment through structured electronic medical record review and direct patient interview. The following variables were recorded: demographics (age at diagnosis), presenting symptoms (dysmenorrhea, abdominal/pelvic pain, irregular vaginal bleeding/spotting, abnormal vaginal discharge/purulent secretions, pelvic mass), symptom duration, menstrual history, and mode of diagnosis.

### Preparation and image acquisition

All female pelvic MRI examinations were carried out using a 3-T MRI scanner (MAGNETOM Vida 3.0 T) equipped with an abdominal phase-controlled surface coil array and a spinal phase-controlled coil array. A glycerine enema (10 mL) was administered 30 min before the scan was initiated to reduce the presence of intestinal gas and feces and to limit the confounding effects of intestinal gas and intestinal contents on the resulting images. The bladder was emptied before the exam. Patients were asked to remove metal objects from their bodies, wear earplugs and earphones to reduce the noise level, adopt a head-first supine position for the scan, and apply pressure on the abdominal band with the external belt to reduce breathing motion artefacts. The scan sequences included 3D T2 (slice thickness 1 mm) imaging, T2 TSE coronal, sagittal, and axial imaging, T1 TSE axial imaging, diffusion-weighted imaging, and large FOV T2 coronal imaging (reaching kidney level). A detailed overview of the sequence parameters is shown in Table 1. Full-spine X-ray examinations were carried out for patients to evaluate the skeletal development.

**Table 1:** Parameters of MRI sequences

Parameters	T2-weighted imaging	T2-weighted imaging	T2-weighted imaging	T2-weighted imaging	T1-weighted imaging	Large FOV T2-weighted imaging
Imaging acquisition, Orientation	TSE Axial	TSE Sagital	TSE Coronal	3D SPACE	TSE Axial	TSE Coronal
Repetition time/echo time	4450/104	4570/104	4490/106	1500/99	516/11	4490/106
FOV	73 × 73	73 × 73	94 × 94	128 × 128	44 × 44	400 × 400
Matrix	128 × 128	128 × 128	128 × 128	128 × 128	128 × 128	352 × 352
Spatial resolution	0.57 × 0.57	0.57 × 0.57	0.74 × 0.74	1.0 × 1.0	0.34 × 0.34	1.14 × 1.14
Slice thickness (mm)	6	5.5	6	1	6	6
Slice gap	1.80	0.55	1.20	6	1.80	1.2
Total imaging time	02:35	02:49	02:10	05:43	02:26	01:07
Fat suppression			Yes			
Sense factor				15		

*TSE* turbo spin echo, *SENSE* sensitivity encoding

### Image analysis

**Fig. 2:**
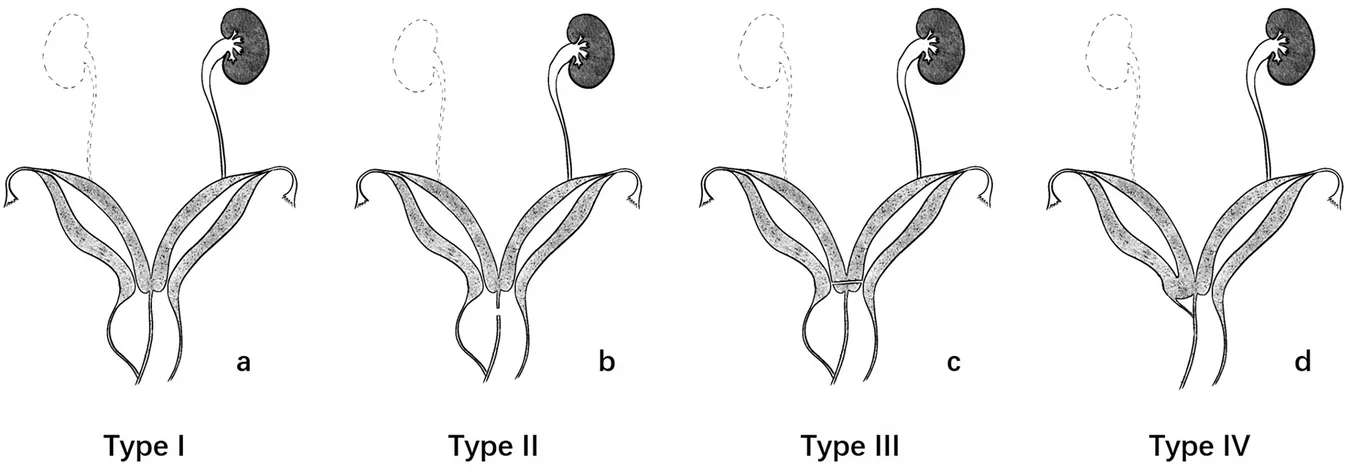
The classification of HWWS. **a**–**d** The subtypes of HWWS syndrome are depicted here

Two radiologists with extensive experience (8 years and 15 years) in gynecological imaging independently evaluated the preoperative MRI images in two separate reading sessions, with surgical outcomes blinded. In the first session, only conventional T2 TSE images (coronal, sagittal, and axial) were provided for evaluation. In the second session, conducted at least 2 weeks later to ensure adequate washout, only high-resolution 3D T2 images were evaluated. The order of the two sessions was randomized for each patient using a computer-generated random sequence. After completion of both sessions, any discrepancies between the two radiologists were arbitrated by a senior radiologist with 20 years of experience in gynecological MRI, who had access to both sequences for consensus. A structured evaluation form was used for each patient in each session, systematically assessing the following features in Table 2. The diagnostic and classification results of HWWS from preoperative MRI were compared with the surgical outcomes to obtain the concordance rate between preoperative MRI diagnosis and surgical results. Uterine anomalies were classified according to the ESHRE (European Society of Human Reproduction and Embryology)/ESGE (European Society for Gynaecological Endoscopy) consensus on congenital uterine anomalies [25] and the American Society for Reproductive Medicine Müllerian Anomalies Classification 2021 (MAC2021) [26, 27]. These systems categorize uterine morphology in HWWS as didelphys, septate, or bicornuate uterus. However, neither system specifically addresses the anatomical heterogeneity of HWWS. We therefore adopted the HWWS-specific classification proposed by Zhu et al [24, 28], which categorizes patients into four types based on the level of obstruction and presence of communications: Type I (imperforate oblique septum without communication) (Fig. 2a), Type II (perforate oblique septum with vaginal communication) (Fig. 2b), Type III (imperforate oblique septum with cervical fistula) (Fig. 2c), and Type IV (unilateral cervical atresia with complete disconnection) (Fig. 2d).

**Table 2:** Structured imaging evaluation criteria

Category	Assessment items
Uterine morphology	Configuration (didelphys/septate/bicornuate); external fundal contour; internal septum (complete/partial/absent)
Cervical assessment	Number of cervices; cervical septum (present/absent); cervical atresia (present/absent; side)
Vaginal assessment	Oblique vaginal septum (present/absent; side); distance from septum distal end to vaginal introitus (cm); vaginal cavity distension
Communication assessment	Vaginal septum fistula (present/absent); cervical fistula/communication (present/absent);
Obstruction assessment	Obstruction level (vaginal/cervical); side of obstruction; intraluminal content
Associated anomalies	ovarian endometriosis; hematosalpinx; adenomyosis; fibroids
Urinary anomalies	Ipsilateral renal agenesis; contralateral renal status;
Skeletal assessment	Spinal curvature classification; Cobb angle (degrees)

Adnexal and other pelvic findings were comprehensively evaluated on T2 TSE, 3D T2, and T1 TSE sequences. Renal abnormalities are assessed on large FOV T2 coronal images that include the abdomen. The spinal development abnormalities were classified as normal (Cobb angle ≤ 10°), slightly scoliosis (10° < Cobb angle ≤ 20°), or scoliosis (Cobb angle ≥ 20°).

### Three-dimensional reconstruction

3D reconstruction with color rendering was selectively performed for complex cases requiring enhanced spatial visualization for surgical planning or patient education, rather than routinely applied to all patients. Three-dimensional reconstruction post-processing examination was ultimately performed on 17 patients. Raw medical images are acquired in DICOM format, then preprocessed (denoising, enhancement, segmentation) to improve 2D visualization. A 3D reconstruction algorithm generates a model from the processed image stack, which can be rendered stereoscopically with interactive functions including zoom, rotation, selection, and pseudo-coloring.

### Surgery

Fifty-two patients received surgical intervention. The surgical methods used were documented. Based on the surgical reports, the degree of obstruction and the communication between the two sides were assessed and compared with the findings from MRI.

### Statistical analysis

Categorical variables are expressed as frequency (percentage). Continuous variables with normal distribution are expressed as mean ± SD (ranges). Inter-reader agreement for categorical imaging features was assessed using Cohen’s kappa coefficient (κ) with 95% confidence intervals (CI). Agreement was interpreted as: poor (κ < 0.20), fair (0.21–0.40), moderate (0.41–0.60), good (0.61–0.80), or excellent (0.81–1.00). The McNemar test was used to compare the detection rates of vaginal septum fistulas between 3D T2 and T2 TSE sequences. The association between HWWS type and adnexal/pelvic findings (ovarian endometriosis, hematosalpinx, uterine adenomyosis, and fibroids) was analyzed using the chi-squared test or Fisher’s exact test, as appropriate. A *p*-value of < 0.05 was considered statistically significant.

## Results

### Patient demographics and clinical presentation

The mean age of onset in the 70 patients was 20.4 ± 5.8 years (range 9–39 years) at diagnosis. Among all patients, 33 (47.1%) presented with dysmenorrhea, 13 (18.6%) presented with abdominal pain, 12 (17.1%) had spotting or irregular vaginal bleeding, 5 (7.1%) presented with abnormal vaginal discharge or purulent secretions, and 7 patients (10.0%) were asymptomatic and diagnosed incidentally.

### Uterine anomalies

The uterus was didelphic in 54 patients (77.1%), septate in 14 patients (20%), and bicornuate in 2 patients (2.9%). The diagnosis of uterine anomaly by T2 3D MRI was in accordance with T2 TSE imaging in 70 patients (100%).

### Obstruction site, communications, and surgical correlation

Among the 70 patients, 65 (92.9%) exhibited varying degrees of obstruction, manifested as hematometra, hematosalpinx, or hydrosalpinx (clinical symptoms are detailed in the preceding paragraph). A total of 46 patients (65.7%) presented with vaginal oblique septum, including 26 (37.1%) on the right side and 20 (28.6%) on the left side. Twenty-four patients (34.3%) showed unilateral cervical atresia, with 11 (15.7%) on the right side and 13 (18.6%) on the left side. A total of 52 patients underwent surgical treatment, serving as the reference standard for obstruction site and communication assessment. The remaining 18 patients did not undergo surgery; their obstruction sites and communications were evaluated by MRI only, with inter-sequence agreement (T2-TSE vs 3D) reported for these cases. The average distance from the vaginal oblique septum to the external vaginal orifice on MRI images was 3.2 ± 1.1 cm (range: 0.6–4.9 cm).

### Surgically confirmed cases (*n* = 52)

The diagnoses of obstruction sites made using MRI T2 TSE images and 3D images were completely consistent with the surgical results in all 52 patients (100%).

#### Vaginal-level obstruction (*n* = 31, 59.6% of surgical patients)

**Fig. 3:**
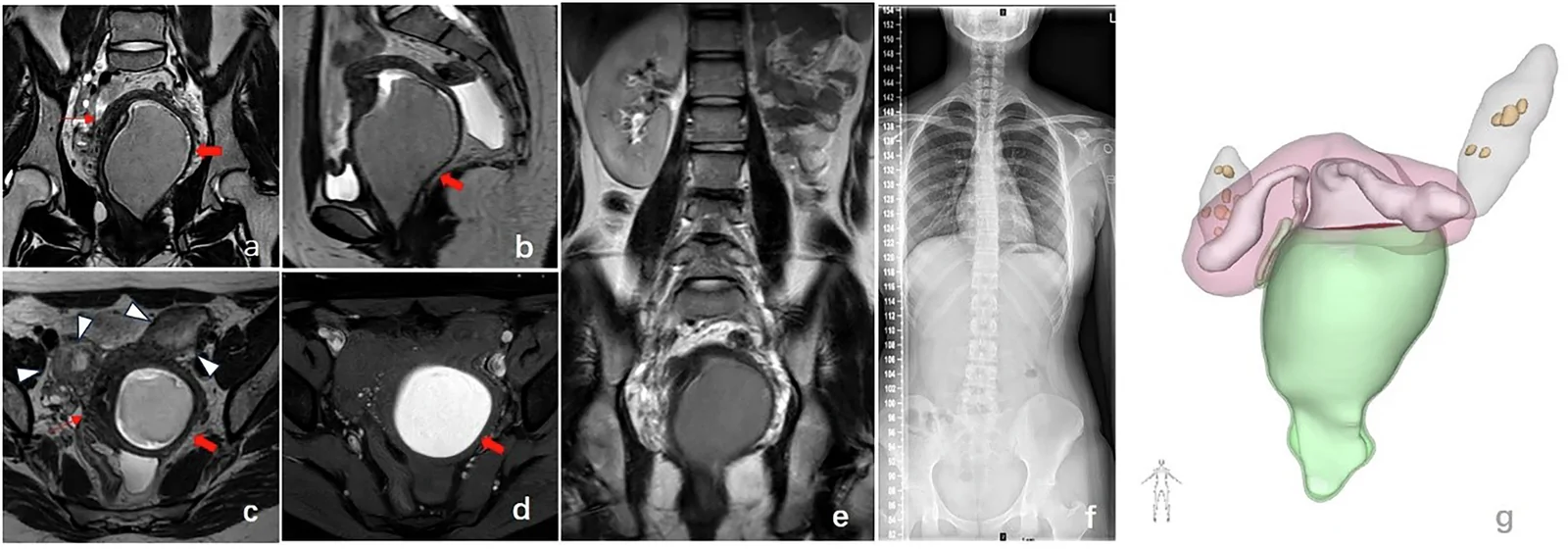
Type I HWWS. A 15-year-old girl with an incidental finding of renal agenesis and uterine malformation. **a**–**c** Coronal (**a**), sagittal (**b**), and axial (**c**) T2-weighted 3D images demonstrate an oblique vaginal septum. The left lower vagina is obstructed and distended with a high T2 signal (thick red arrow), while the right vagina is collapsed and flattened (thin red arrow). **c** Axial image also shows a didelphic uterus (white arrow heads). **d** T1-weighted image reveals high T1-signal blood products within the distended left vagina. **e** Large-FOV coronal T2 image demonstrates the absence of the left kidney. **f** Spinal radiograph shows mild scoliosis. **g** 3D reconstructed model depicts the dilated left vagina (green) and the didelphic uterine anomaly (pink). The patient underwent excision of the oblique vaginal septum; surgery confirmed an oblique septum with a didelphic uterus, consistent with a Type I HWWS

**Fig. 4:**
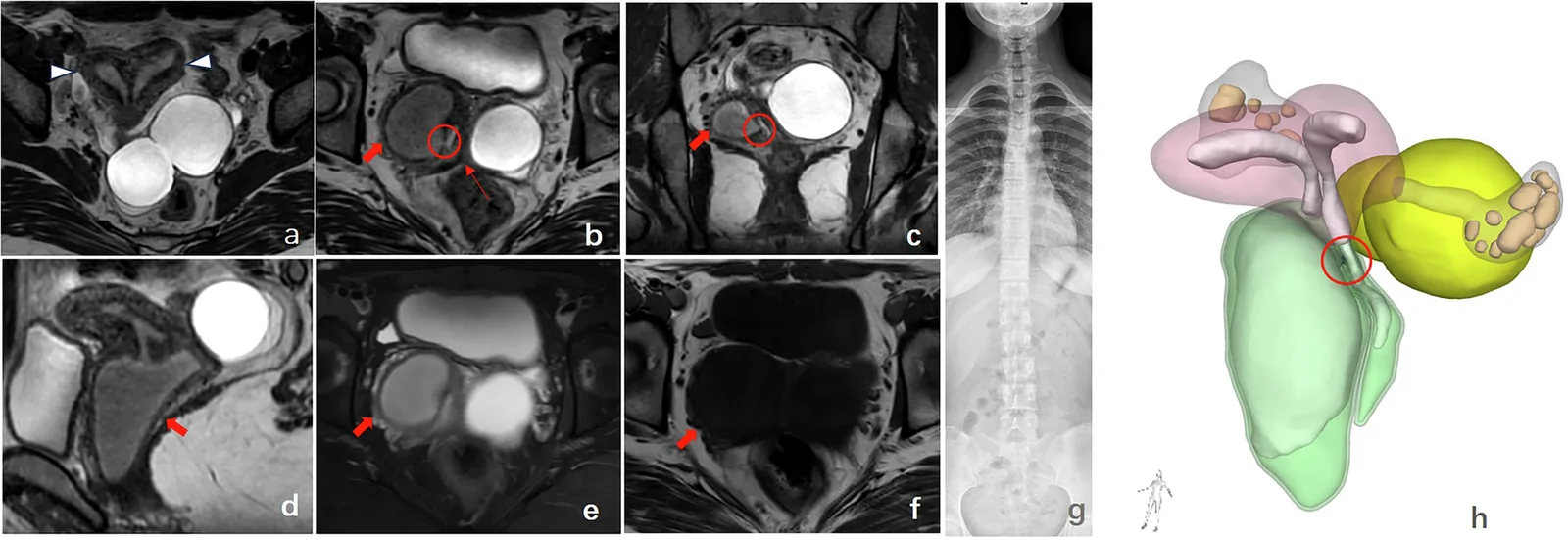
Type II HWWS. A 15-year-old girl presented with purulent vaginal discharge and abdominal pain. **a** Axial T2-weighted 3D image demonstrates a complete septate uterus (white arrow heads). **b**–**d** Axial (**b**), coronal (**c**), and sagittal (**d**) T2-weighted 3D images show obstruction of the lower right vagina, with marked T2-hyperintense distension of the right vagina (thick red arrow). A fistula in the oblique septum communicating with the contralateral vagina is visible (red circle). **e** T2-weighted TSE sequence confirms the distended right vagina and the oblique septum, but the fistula is not clearly depicted. **f** T1-weighted TSE image shows T1-hypointense fluid within the dilated vagina. **g** Whole-spine radiograph reveals mild scoliosis. Right renal agenesis was also present (image not shown). **h** 3D reconstructed model illustrates the dilated right vagina (green), the fistula in the oblique septum (red circle), the complete septate uterus (pink), and a cystic lesion posterior to the uterus (yellow). The patient underwent excision of the oblique vaginal septum; surgery confirmed the oblique septum and the communication, consistent with a Type II HWWS

**Fig. 5:**
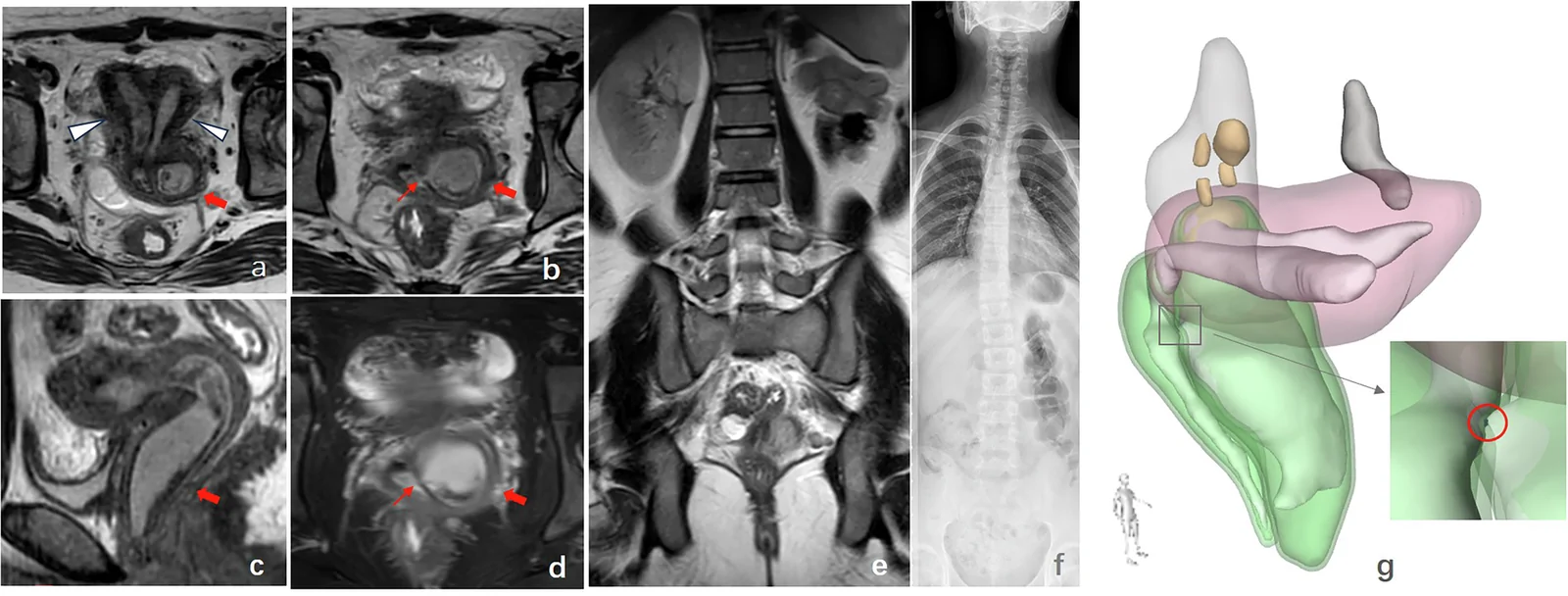
Type II HWWS. A 12-year-old girl presented with prolonged menstruation. **a** Axial T2-weighted 3D image reveals a complete septate uterus (white arrow heads). **b**, **c** Axial (**b**) and sagittal (**c**) T2-weighted 3D images demonstrate an obstructed and distended left vagina, and a fistula in the oblique septum communicating with the contralateral vagina (thin red arrow). **d** T2-weighted TSE sequence confirms the dilated left vagina and the septum, but the fistula is not clearly visualized (thin arrow). **e** Large-FOV coronal T2 image shows the absence of the left kidney. **f** Whole-spine radiograph shows mild scoliosis. **g** 3D reconstructed model depicts the distended left vagina (green), the fistula in the oblique septum (red circle), and the complete septate uterus (pink). The patient underwent excision of the oblique vaginal septum. Surgery confirmed the oblique septum and its fistula, consistent with a Type II HWWS

All 31 underwent resection of the vaginal oblique septum. Of these, 18 (34.6%) had no fistulas detected at surgery (Type I) (Fig. 3), 11 cases (21.2%) had vaginal septum fistulas confirmed surgically (Type II) (Figs. 4 and 5), and 2 (3.8%) cases had cervical communications confirmed (Type III) (Supplementary Fig. 1). MRI T2 TSE images identified no cases (0%) of vaginal septum fistulas preoperatively, whereas 3D T2 identified 7 cases (63.6%) of vaginal septum fistulas preoperatively (*p* = 0.005). The two cases of cervical communication were identified by both sequences preoperatively.

#### Cervical-level obstruction (*n* = 21, 40.4% of surgical patients)

**Fig. 6:**
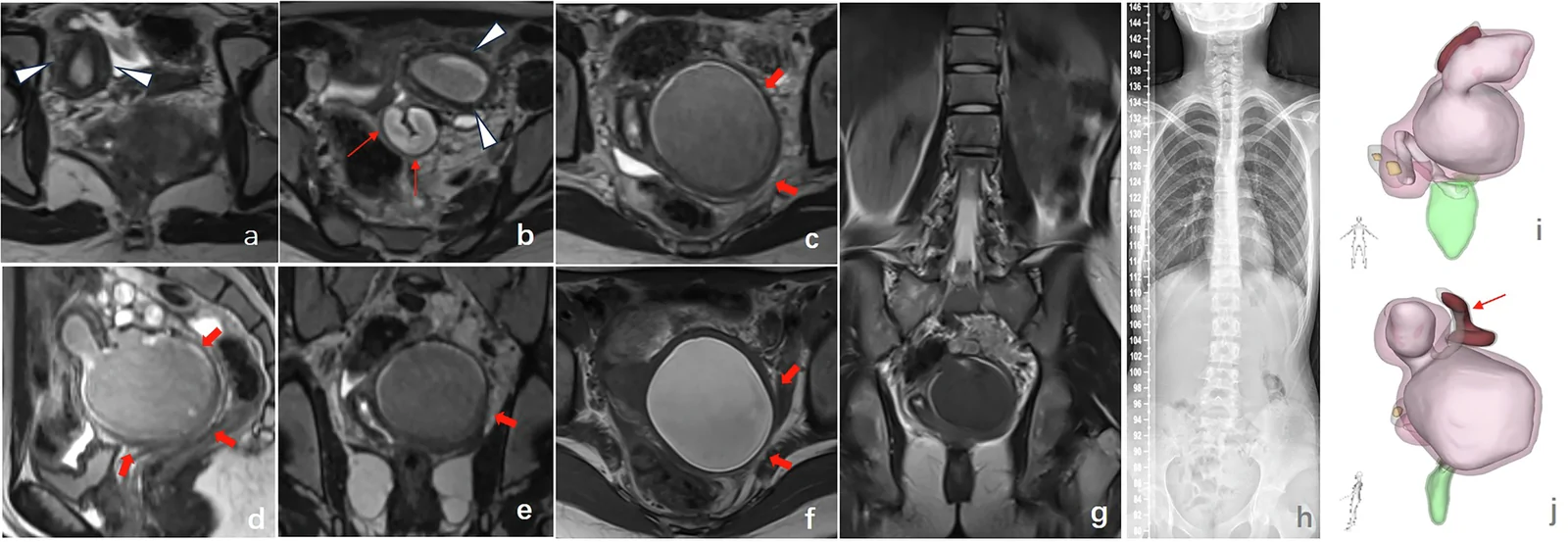
Type IV HWWS. A 14-year-old girl presented with dysmenorrhea. **a**, **b** Axial T2-weighted 3D images demonstrate a didelphic uterus (white arrow heads) and a tortuous, dilated left fallopian tube (thin red arrows). **c**–**e** Axial (**c**), sagittal (**d**), and coronal (**e**) T2-weighted 3D images reveal a cervical septum with atresia of the left external cervical os and marked distension of the left cervical canal (thick red arrows). **f** T1-weighted TSE image shows high-T1-signal blood products within the dilated cervical canal. **g** Large-FOV coronal T2 image confirms absence of the left kidney. **h** Whole-spine radiograph reveals mild scoliosis. **i**, **j** 3D reconstruction illustrates the markedly dilated left cervix (pink) and the blood-filled, dilated left fallopian tube (dark red, thin red arrow). The patient underwent left hemi-hysterectomy with ipsilateral salpingectomy; surgery confirmed Type IV HWWS

All 21 underwent ipsilateral hysterectomy. Of these, 19 cases (36.5%) had no cervical septum fistulas at surgery (Type IV) (Fig. 6), and 2 cases (3.8%) had cervical communication confirmed surgically (special Type III + IV) (Supplementary Fig. 2). Both cases of cervical communication were identified by the 3D imaging and T2 TSE.

### Non-surgical patients (*n* = 18)

MRI evaluation (without surgical confirmation) suggested vaginal-level obstruction in 15 (83.3%) and cervical-level obstruction in 3 (16.7%). Inter-sequence agreement was as follows: both 3D and T2 TSE identified 3 cases of cervical communication (Type III); 3D MRI additionally identified 3 cases of vaginal septum fistula (Type II) that were not detected on T2 TSE. These findings were not confirmed by gynecological examination.

### Adnexal and other pelvic findings

Ovarian endometriosis was diagnosed in 16 patients (22.9%), including 3 (11.1%) with type I, one (7.1%) with type II, and one (20%) with type III, one (50%) with type III + IV, and 10 (45.5%) with type IV (*p* = 0.008). Hematosalpinx was identified in 16 patients (22.9%), including 5 (18.5%) with type I, one (7.1%) with type II, and one (20%) with type III, one (50%) with type III + IV, and 8 (36.4%) with type IV (*p* = 0.049). And 10 patients (14.3%) showed polycystic ovary-like changes.

Uterine adenomyosis was diagnosed in 8 patients (11.4%): 3/27 (11.1%) with Type I and 5/22 (22.7%) with Type IV (*p* = 0.007). Uterine fibroids were diagnosed in 3 patients (4.3%): all were in Type IV patients (*p* = 0.007). Fourteen cases (20%) had cystic lesions in the cervix or paravaginal area, which were considered to be remnants of the Müllerian duct or Gartner’s cysts. Inter-reader agreement for adnexal and pelvic findings was good to excellent (ovarian endometriosis: κ = 0.82; hematosalpinx: κ = 0.85; adenomyosis: κ = 0.79).

### Urinary and skeletal system

Sixty-nine patients (98.6%) had renal agenesis on the obstructed side. Among them, one case had a duplicated kidney on the non-obstructed side. One patient (1.4%) did not have any urinary malformations. Eighteen patients (25.7%) had scoliosis (Cobb angle ≥ 20°), 29 patients (41.4%) exhibited a slight scoliosis (10° < Cobb angle < 20°), and 23 patients (32.9%) showed no obvious spinal developmental abnormalities (Cobb angle ≤ 10°).

### Inter-reader agreement

Inter-reader agreement for T2 TSE sequences was excellent for uterine morphology classification (κ = 0.89, 95% CI: 0.80–0.98) and obstruction site determination (κ = 0.85, 95% CI: 0.74–0.96). Agreement for vaginal septum fistula detection on T2 TSE was poor (κ = −0.10), reflecting the limited sensitivity of this sequence. For 3D T2 sequences, inter-reader agreement was excellent for uterine morphology (κ = 0.92, 95% CI: 0.84–1.00), obstruction site (κ = 0.88, 95% CI: 0.78–0.98), and good for vaginal septum fistula detection (κ = 0.76, 95% CI: 0.58–0.94). Agreement for cervical communication detection was excellent on both sequences (κ = 1.00), given the limited number of positive cases. The ICC for septum-to-introitus distance measurement was 0.89 (95% CI: 0.82–0.94) on 3D T2. All discrepancies were resolved by the senior radiologist’s arbitration.

## Discussion

In this study, we prospectively enrolled 70 patients clinically diagnosed with HWWS syndrome. We used both conventional MRI sequences and 3D thin-slice high-resolution sequences to evaluate the urogenital system of patients with HWWS. Additionally, we comprehensively assessed the overall condition of the patients by combining spinal X-ray examinations and other tests. Among these patients, 52 eventually underwent surgical treatment. Our study found that MRI plays an important role in the preoperative diagnosis and classification of HWWS. The 3D sequences can provide clearer and more precise information, which is helpful for classification and diagnosis.

The typical manifestations of HWWS include the “triple triad” of didelphys uterus, unilateral vaginal oblique septum, and unilateral renal agenesis. The anatomical characteristics of HWWS exhibit heterogeneity. Uterine anomalies may include double uterus, bicornuate uterus, and septate uterus; cervical anomalies may present as double cervix, cervical septum, underdeveloped or even obstructed cervix on one side; vaginal septa can be classified as either perforate or non-perforate; and the septate side and the healthy side may have communication at different levels [29].

Accurate preoperative classification of HWWS is essential for surgical planning. In this study, we applied the Zhu et al classification [24, 28], which specifically addresses the spectrum of obstruction and communication patterns seen in HWWS and directly links anatomical subtype to surgical approach. Our results demonstrate that high-resolution 3D T2 MRI enables accurate classification by reliably identifying the obstruction level and detecting small communications that determine subtype designation. For patients with vaginal oblique septum (Types I, II, and III), the most commonly used surgical method is vaginal oblique septum resection. For patients with cervical atresia (Type IV), laparoscopic or transabdominal hysterectomy of the affected side is recommended [30].

Compared to ultrasonography, MRI sequences offer significant advantages in the evaluation of uterine anomalies, the location of oblique septa, the site of obstruction, and the presence of hematometra. In this study, the uterine manifestations included not only didelphys uterus but also septate uterus and bicornuate uterus. Both the T2-weighted turbo spin-echo (TSE) sequences and the 3D sequences demonstrated consistent accuracy in assessing uterine morphology and identifying the sites of obstruction, which were corroborated by surgical findings. These results are in accordance with the conclusions of previous studies.

In previous studies, MRI has had certain limitations in identifying communication between the vagina and cervix, making it difficult to differentiate between Type I, Type II, and Type III conditions [17]. In this study, the 3D thin-slice sequence with a resolution of 1 mm was used. Compared to the T2-weighted turbo spin-echo (TSE) sequence, the 3D thin-slice sequence significantly improved the preoperative diagnostic rate of small holes in the vaginal oblique septum (7/11, 63.6%) and accurately identified cervical communication (2/2), thereby enhancing the accuracy of classification in patients with vaginal obstruction. We attribute this discrepancy to several technical factors. First, the slice thickness of conventional T2-TSE sequences (4–6 mm) exceeds the diameter of most vaginal septum fistulas, rendering them susceptible to partial volume averaging. Second, the vaginal cavity is often collapsed or compressed by adjacent hematocolpos, further obscuring small communications on thick-slice imaging. Third, 3D sequences provide isotropic 1-mm resolution with multiplanar reformatting capability, enabling visualization of fistulous tracts in arbitrary planes. From a clinical perspective, failure to identify a vaginal septum fistula on T2-TSE may lead to misclassification of Type II HWWS as Type I. Therefore, our findings underscore that reliance on conventional T2-TSE alone carries a risk of underestimating communications in HWWS, and support the routine addition of high-resolution 3D sequences to the imaging protocol.

Clinically, patients with Type I often present with dysmenorrhea as the main complaint and have an earlier age of onset [30]. Type II and Type III patients (especially Type II) mainly present with bloody or purulent vaginal discharge, typically after reaching sexual maturity during adolescence [30]. Type I patients have complete obstruction. In this study, it was also found that compared to Type II, Type I patients are more likely to have associated conditions such as tubal hematometra, ovarian endometriosis, and adenomyosis, which require earlier diagnosis and surgical treatment. Therefore, the use of 3D MRI for accurate preoperative classification can aid clinical decision-making and treatment planning. Moreover, in patients who did not undergo surgery, 3D MRI also detected communication between the cervix and vaginal oblique septum that was not identified by physical examination, which is highly informative for clinicians.

In previous studies, the proportion of Type IV patients was relatively low [17, 28]. In this study, however, the proportion of Type IV patients was higher (24/70, 34.3%), and even higher among patients who underwent surgery (19/52, 36.5%). This is related to the fact that Type IV is often difficult to diagnose definitively in other hospitals.

As shown in Table 3b, Type IV patients exhibited a significantly higher prevalence of ovarian endometriosis (10/16, 62.5%; *p* = 0.008) and hematosalpinx (8/16, 50%; *p* = 0.049) compared to other types, and also showed a higher rate of adenomyosis (5/8, 62.5%; *p* = 0.007). These statistical findings, presented in the “Results” section, suggest that Type IV patients may represent a more severe phenotypic spectrum requiring earlier identification and intervention. The ability of 3D MRI to accurately identify cervical atresia and obstruction preoperatively is of great significance for guiding the diagnosis and treatment of patients.

**Table 3:** Obstruction sites, communications, and surgical correlation by HWWS type (**a**); associated findings by HWWS type (**b**)

a				
HWWS type	Surgical patients (*n* = 52)	Non-surgical patients (*n* = 18)	Communication detected by 3D T2	Communication detected by T2 TSE
Type I	18 (34.6%)	9	0/18	0/18
Type II	11 (21.2%)	3	7/11 (63.6%)	0/11 (0%)
Type III	2 (3.8%)	3	2/2 (100%)	2/2 (100%)
Type IV	19 (36.5%)	3	N/A	N/A
Type III + IV	2 (3.8%)	0	2/2 (100%)	2/2 (100%)

b					
HWWS type	*n*	Ovarian endometriosis	Hematosalpinx	Adenomyosis	Fibroids
Type I	27	3 (11.1%)	5 (18.5%)	3 (11.1%)	0 (0%)
Type II	14	1 (7.1%)	1 (7.1%)	0 (0%)	0 (0%)
Type III	5	1 (20.0%)	1 (20.0%)	0 (0%)	0 (0%)
Type IV	22	10 (45.5%)	8 (36.4%)	5 (22.7%)	3 (13.6%)
Type III + IV	2	1 (50.0%)	1 (50.0%)	0 (0%)	0 (0%)
*p*-value	-	0.008	0.049	0.007*	0.007*

* Fisher’s exact test

Unilateral renal dysplasia is also the most common associated anomaly of HWWS, with the most frequent manifestation being renal agenesis or hypoplasia on the obstructed side. The incidence of renal agenesis has been reported in the literature to range from 74% to 97.7% [8, 28, 31]. In this study, we incorporated a large field-of-view T2-weighted coronal imaging sequence into the MRI scan to evaluate the development of the urinary system, which allows for a more complete and comprehensive assessment of the urinary tract. In our study, the incidence of renal agenesis on the obstructed side was 98.6% (69/70). The presence of renal agenesis on the obstructed side is particularly helpful for the diagnosis of vaginal oblique septum syndrome.

Patients with genital tract malformations often also have spinal developmental abnormalities [28]. A multicenter retrospective study published in 2025 that included 415 cases of HWWS reported a 15.1% incidence of associated skeletal system malformations, with more than half of these patients having scoliosis [20]. In this study, we used full-spine X-ray to assess the skeletal development of the patients and scoliosis was quantified by the Cobb angle, which allows for a more comprehensive and precise evaluation of the skeletal condition. In our study, the incidence of significant scoliosis in HWWS patients was 25.7% (18/70). We further distinguished scoliosis from mild scoliosis (29/70, 41.4%); the latter requires only surveillance and no clinical intervention. This provides a relatively large sample size and more comprehensive data for the diagnosis and treatment of HWWS patients.

This study also has several limitations. First, all patients had emptied their bladders before the examination and used rectal suppositories to suppress intestinal motility. However, due to the longer scanning time of the 3D sequences, some patients still exhibited noticeable small bowel motility during the scan. Additionally, the size of the bladder may change before and after the examination. In the future, as the scanning protocol becomes more refined, we will consider appropriately reducing the scanning time. Second, although the 3D sequences have already shown significant advantages in identifying communication between the vaginal oblique septum and the cervix in this study, the vaginal cavity in some patients was relatively collapsed, which still affected the identification of communication through the vaginal oblique septum. In the future, with the informed consent of patients, we will consider using methods such as vaginal gel injection to improve the resolution of vaginal structures. Third, a small number of patients in this study did not undergo surgical treatment and therefore could not provide accurate data on the site of obstruction and the presence of communication. However, to the best of our knowledge, this study remains the largest prospective case series on HWWS to date. In the future, we look forward to having more HWWS patients complete surgical treatment.

In conclusion, high-resolution MRI T2 3D sequences demonstrate greater advantages over T2 TSE sequences in the preoperative diagnosis and classification of HWWS patients, particularly in identifying the site of obstruction and cervicovaginal communications. This approach provides more accurate and effective guidance for selecting the optimal surgical timing and planning the surgical strategy for HWWS patients. Combined with large-field T2-weighted fast coronal imaging and spinal X-rays, it enables a comprehensive evaluation of the condition in HWWS patients.

## Supplementary information

ELECTRONIC SUPPLEMENTARY MATERIAL

## Data Availability

The datasets generated and/or analyzed during the current study are not publicly available due to patient privacy concerns. However, de-identified data may be made available from the corresponding author upon reasonable request, subject to approval by the relevant ethics committee.

## Abbreviations

3D: Three-dimensional
ESGE: European Society for Gynaecological Endoscopy
FOV: Field of view
HWWS: Herlyn-Werner-Wunderlich syndrome
MRI: Magnetic resonance imaging
